# Pharmacological characterisation of murine α4β1δ GABA_A_ receptors expressed in *Xenopus* oocytes

**DOI:** 10.1186/s12868-015-0148-4

**Published:** 2015-03-05

**Authors:** Inge S Villumsen, Petrine Wellendorph, Trevor G Smart

**Affiliations:** Department of Neuroscience, Physiology & Pharmacology, University College London, Gower Street, London, WC1E 6BT UK; Department of Drug Design and Pharmacology, Faculty of Health and Medical Sciences, University of Copenhagen, Fruebjergvej 3, 2100 Copenhagen, Denmark

**Keywords:** γ-aminobutyric acid, GABA_A_ receptors, α4β1δ subtype, Extrasynaptic receptors, β1 subunit

## Abstract

**Background:**

GABA_A_ receptor subunit composition has a profound effect on the receptor’s physiological and pharmacological properties. The receptor β subunit is widely recognised for its importance in receptor assembly, trafficking and post-translational modifications, but its influence on extrasynaptic GABA_A_ receptor function is less well understood. Here, we examine the pharmacological properties of a potentially native extrasynaptic GABA_A_ receptor that incorporates the β1 subunit, specifically composed of α4β1δ and α4β1 subunits.

**Results:**

GABA activated concentration-dependent responses at α4β1δ and α4β1 receptors with EC_50_ values in the nanomolar to micromolar range, respectively. The divalent cations Zn^2+^ and Cu^2+^, and the β1-selective inhibitor salicylidine salicylhydrazide (SCS), inhibited GABA-activated currents at α4β1δ receptors. Surprisingly the α4β1 receptor demonstrated biphasic sensitivity to Zn^2+^ inhibition that may reflect variable subunit stoichiometries with differing sensitivity to Zn^2+^. The neurosteroid tetrahydro-deoxycorticosterone (THDOC) significantly increased GABA-initiated responses in concentrations above 30 nM for α4β1δ receptors.

**Conclusions:**

With this study we report the first pharmacological characterisation of various GABA_A_ receptor ligands acting at murine α4β1δ GABA_A_ receptors, thereby improving our understanding of the molecular pharmacology of this receptor isoform. This study highlights some notable differences in the pharmacology of murine and human α4β1δ receptors. We consider the likelihood that the α4β1δ receptor may play a role as an extrasynaptic GABA_A_ receptor in the nervous system.

## Background

GABA_A_ receptors are pentameric ligand-gated ion channels that mediate phasic and tonic inhibition in the central nervous system [1]. When activated by GABA the integral ion channel opens, rendering the receptor permeable to chloride ions. The co-assembly of combinations of 19 different subunits (α1-6, β1-3, γ1-3, δ, ε, θ, π and ρ1-3) into homo- or hetero-pentameric receptors results in multiple different GABA_A_ receptor isoforms with different function, pharmacology and neuronal location [2-4]. The stoichiometry of the majority of these receptors is considered to be 2α:2β:1γ/δ, predominantly arranged as β-α-β-α-γ/δ in an anticlockwise manner when viewed from the extracellular space [5-7]. However, the arrangement for δ subunit-containing receptors seems to be dependent on the experimental conditions [6,8-12].

Receptors incorporating α1, α2 or α3 subunits most commonly assemble with β and γ subunits, and are generally located at the synapse where they mediate phasic inhibition; whereas α4 and α6-containing receptors assemble with β and δ subunits, and are found extrasynaptically, mediating tonic inhibition [13]. The δ subunit is found almost exclusively in the extrasynaptic domain and forms α4βδ or α6βδ receptors in the cortex and cerebellum, respectively [2]. However, it has also been suggested that δ can co-assemble with α1 subunits in hippocampal interneurons [14,15].

The existence of α4β2/3δ receptors in brain regions such as the hippocampal dentate gyrus is well established, but there is a paucity of data which characterises the potential native expression of the α4β1δ receptor [2]. This is largely due to the limited number of β1-subtype-selective compounds available [16], and the fact that no β1 subunit knock-out mouse has been generated. As a consequence, most previous studies investigating α4βδ receptors have focused on those subtypes containing the β2 or β3 subunits as these are confirmed to be native receptor combinations [2,17,18]. However, co-regulation of the expression levels of α4, β1 and δ subunits in, for example, the periaqueductal grey matter throughout the female oestrous cycle has been reported, suggesting that α4β1δ receptors may perform a hitherto overlooked important physiological role [19]. Also, experimental evidence for the co-expression of α4, β1 and δ subunits at extrasynaptic sites exists for pyramidal hippocampal neurons [20]. Furthermore, recent studies have emphasized the importance of β1-containing receptors in relation to sleep control [21] and ataxia [22], and polymorphic mutants of the β1 subunit are thought to be important in determining the extent of alcohol consumption [23]. Therefore, since α4, β1 and δ subunits are co-expressed in several brain regions [24], the possibility exists that these subunits can co-assemble into native functional receptors, most likely at extrasynaptic sites, raising the prospect that such a receptor subtype may be important for neuronal physiology.

Few studies have investigated the properties of either human [25-27] or murine [23,28] recombinant α4β1δ receptors, and none of these have fully addressed the characterisation of classic GABA_A_ receptor ligands. In these studies, interesting pharmacological differences between murine and human α4β1δ receptors are manifest: human receptors expressed in *Xenopus* oocytes [25] are constitutively active and display a GABA EC_50_ in the mid-nanomolar range whereas the rat/mouse receptor expressed in oocytes or HEK293 cells display low-micromolar sensitivity towards activation by GABA [23,28]. To address this apparent species-dependent pharmacology and to address the effects of commonly used GABA receptor ligands, we report the pharmacological profiles for several GABA_A_ receptor ligands at recombinant murine α4β1 and α4β1δ receptors expressed in *Xenopus laevis* oocytes.

## Results

### GABA concentration response relationships for α4β1 and α4β1δ receptors

**Figure 1:**
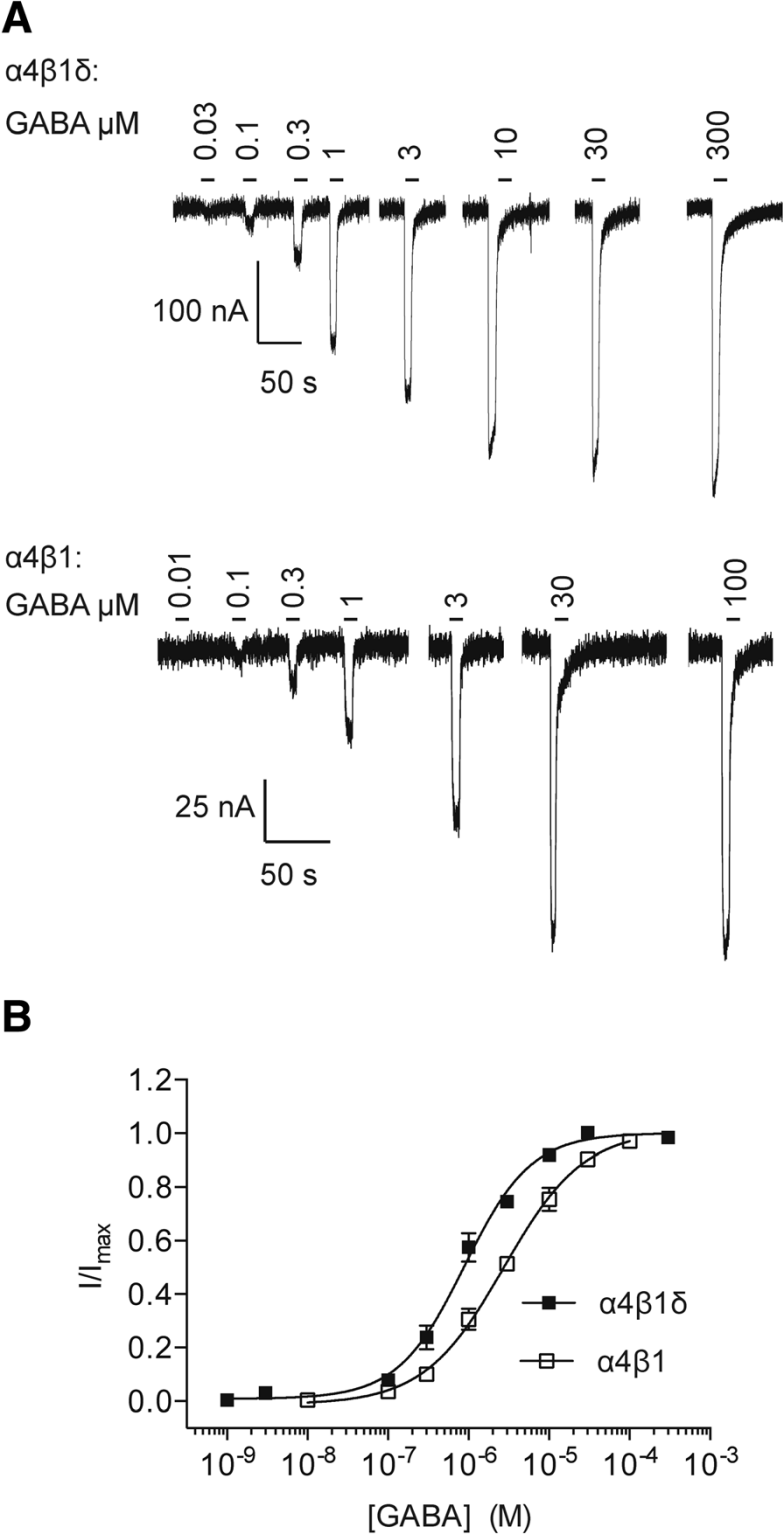
**Examples of GABA-activated currents recorded from cDNA-injected** ***Xenopus*** **oocytes expressing α4β1δ and α4β1 receptors. A**, Representative membrane currents for α4β1δ receptors (upper panel) and α4β1 receptors (lower panel) in response to increasing concentrations of GABA. The oocytes were voltage clamped at -60 mV. **B**, GABA concentration response curves for α4β1δ (n = 6) and α4β1(n = 5) receptors. All data points represent means ± SEMs.

GABA concentration response curves were generated for α4β1 and α4β1δ receptors to evaluate GABA potency at these receptors. Oocytes were challenged with increasing concentrations of GABA (Figure 1A). Depending upon expression levels, maximal currents to GABA ranged from 100 to 3000 nA for cells expressing α4β1 receptors and from 300 to 3000 nA for cells expressing α4β1δ receptors. The holding currents were between 0 and -40 nA and the receptors were not constitutively active. The GABA EC_50_ values were 0.89 μM for α4β1δ receptors and 2.7 μM for α4β1 receptors (Figure 1B, Table 1). GABA concentration-response data were fitted as monophasic curves, with Hill coefficients of 1.1 and 0.87 for α4β1δ and α4β1 receptors, respectively (Table 1).

**Table 1:** **Pharmacological profiles of GABA**_A_ **ligands at α4β1 and α4β1δ receptors**

	**EC**_50_ **(μM) (pEC**_50_ **± SEM)**	**IC**_50_ **(μM) (pIC**_50_ **± SEM)**	**n**_H_ **(± SEM)**	**N**
**GABA**				
α4β1δ	0.89 (6.05 ± 0.05)		1.1 ± 0.11	**6**
α4β1	2.7 (5.56 ± 0.05)		0.87 ± 0.079	**5**
**Zn** ^2+^				
α4β1δ		3.3 (5.48 ± 0.03)	−0.75 ± 0.041	**6**
α4β1^a^		*IC*_50A_		**6**
		0.00013 (9.87 ± 0.22)	ND	
		*IC*_50B_		
		0.056 (7.25 ± 0.13)	-1.1 ± 0.13	
**Cu** ^2+^				
α4β1δ		0.82 (6.09 ± 0.04)	−1.2 ± 0.13	**12**
**SCS**				
α4β1δ		0.0042 (8.37 ± 0.25)		**4**

Potencies for GABA, Zn^2+^, Cu^2+^ and SCS at α4β1 and α4β1δ receptors are listed. For Zn^2+^ and Cu^2+^, inhibition was measured at the GABA EC_75_. For SCS, inhibition was measured at the GABA EC_20_. ND, not determined. ^a^ Data fit a two-site model (IC_50A_ & IC_50B_) better than a one-site model as determined by an F-test (p < 0.0001).

### Inhibition of GABA responses by divalent cations

Zn^2+^ has been shown to be a useful pharmacological tool to distinguish αβ receptors from αβδ/γ receptors in recombinant expression systems, as δ/γ subunit-containing GABA_A_ receptors are less sensitive to inhibition by Zn^2+^ compared to their αβ counterparts [29,30]. The increased potency at αβ receptors is attributed to the incorporation of a third β subunit that contains an important histidine residue for coordinating Zn^2+^ ions at the top of channel lining M2 region [30,31].

**Figure 2:**
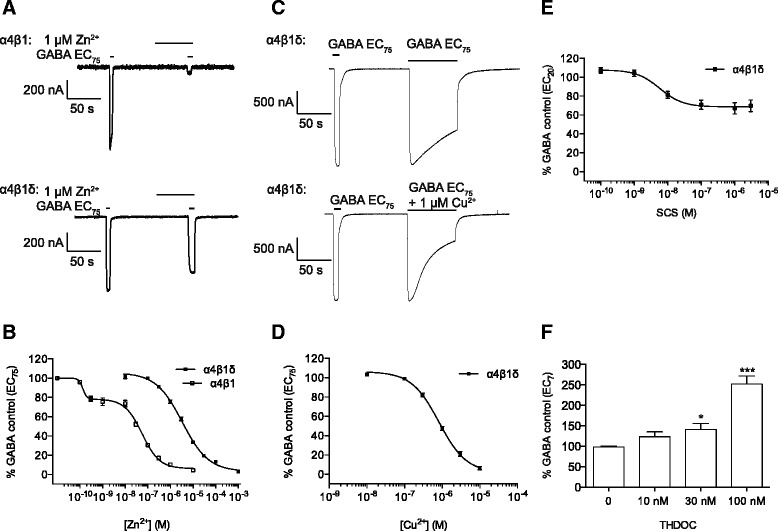
**Pharmacological modulation of GABA responses at α4β1δ receptors by various inhibitors and the neurosteroid, THDOC. A**, Representative membrane currents showing inhibition of GABA (EC_75_) by 1 μM Zn^2+^ at α4β1 (upper) and α4β1δ (lower) receptors. **B**, Zn^2+^ concentration-inhibition relationships for α4β1 (n = 6) and α4β1δ (n = 6) receptors. **C**, Representative currents showing the degree of desensitization when activated by EC_75_ GABA in the absence (upper) and presence (lower) of 1 μM Cu^2+^. **D**, Cu^2+^ concentration-inhibition relationship for GABA EC_75_ desensitized responses at α4β1δ receptors by increasing concentrations of Cu^2+^ (n = 12). **E**, SCS concentration-inhibition relationship for GABA EC_20_ peak responses at α4β1δ receptors by increasing concentrations of SCS (n = 4). **F**, Response of α4β1δ receptors to increasing concentrations of THDOC co-applied with an EC_7_ GABA concentration. The responses were normalised to a preceding application of GABA EC_7_ in the absence of THDOC (n = 9). All data shown are means ± SEMs.

We evaluated Zn^2+^-inhibition of the GABA response for both α4β1 and α4β1δ receptor isoforms to investigate whether Zn^2+^ can distinguish between these receptor subtypes. Oocytes were pre-incubated for 1 min with various concentrations of Zn^2+^ followed by co-application of Zn^2+^ and GABA at an EC_75_ (3 μM for α4β1δ and 8 μM for α4β1). As expected, Zn^2+^ exhibited a higher potency at α4β1 than α4β1δ receptors. For α4β1δ receptors, Zn^2+^ inhibited the GABA response with an IC_50_ of 3.3 μM (Figure 2A,B, Table 1). By contrast, for α4β1 receptors, biphasic inhibitory behaviour was observed with a high potency component (IC_50A_ = 0.13 nM) accounting for ~25% of the total inhibition, and a more dominant lower potency component (IC_50B_ = 56 nM) accounting for the remaining (~75%) inhibition (Figure 2A,B, Table 1).

Cu^2+^ is another well-established inhibitor of GABA currents at γ subunit-containing GABA_A_ receptors [32], but its inhibitory profile at δ subunit-containing receptors has only recently been explored [33]. This revealed that Cu^2+^ was far more potent as an inhibitor of steady-state GABA responses at α6β3δ receptors (IC_50_ = 65 nM) compared to α1β2γ2 receptors (IC_50_ = 85 μM) expressed in human embryonic kidney 293 (HEK293) cells [33]. To further investigate the inhibitory selectivity of Cu^2+^ at α4β1δ receptors, we co-applied increasing concentrations of Cu^2+^ with GABA (EC_75_: 3 μM) for 70 s, and measured the steady-state responses, reproducing the experimental conditions used by McGee et al. [33] to determine the IC_50_ values. This response was taken as the fraction of the peak response of a previously-applied GABA concentration, and normalized to the steady-state response of GABA EC_75_ in the absence Cu^2+^ (Figure 2C). An almost complete inhibition of the α4β1δ GABA-activated response was observed with increasing concentrations of Cu^2+^ (IC_50_ = 0.82 μM) (Figure 2D, Table 1).

### Inhibition by the β1 subunit-selective blocker SCS

The salicylic acid derivative, SCS, is one of only a few compounds known to selectively target β1 subunit-containing GABA_A_ receptors over β2- and β3-containing receptors [34]. SCS is reported to inhibit GABA-activated responses of α2β1γ2, α2β1γ1 and α2β1γ1θ receptors, but the pharmacological profile of SCS at α4 or δ-containing receptors, has not been addressed. To examine the interaction of SCS with α4β1δ receptors, we evaluated the inhibition of the GABA EC_20_ response following pre-application of the antagonist. In this instance, a low EC_20_ concentration was chosen to enable a direct comparison between our study and that obtained previously [34]. SCS inhibited the GABA response with an IC_50_ of 4.2 nM, but did not cause complete inhibition of the GABA current, attaining a steady-state level of inhibition at 67.2 ± 3.2% of control (Figure 2E, Table 1).

### Neurosteroid potentiation at α4β1δ receptors

Neurosteroids are potent modulators at most, if not all, GABA_A_ receptor isoforms [35]. Some studies report that neurosteroids are more potent at recombinant δ subunit-containing receptors compared to their γ subunit-containing counterparts [17,36,37]. Accordingly, we evaluated the sensitivity of α4β1δ receptors to neurosteroid-induced potentiation. Increasing concentrations of THDOC were co-applied with an approximately EC_7_ GABA concentration. A significant increase in the GABA peak-response was recorded with THDOC concentrations of 30 and 100 nM (P < 0.05, one-way analysis of variance (ANOVA) with Dunnett’s post test, Figure 2F).

## Discussion

In this study we investigated the pharmacological profiles of several GABA_A_ receptor ligands at murine α4β1δ receptors. The GABA potency of 0.89 μM (EC_50_) at α4β1δ receptors determined in this study is similar to that previously reported for the rat receptor (2.02 μM) expressed in *Xenopus* oocytes [28], and the mouse/rat receptor (1.99 μM) expressed in HEK293 cells [23]. However, a much higher GABA potency (EC_50_ of 24 nM) has been reported for the human α4β1δ receptor expressed in *Xenopus* oocytes [25]. The GABA potency for human α4β1 (0.72 μM) is reported to be somewhat lower [25] than the murine combination studied here. Furthermore, constitutive activity in the absence of GABA was reported for the human recombinant α4β1δ receptor [25], but this was not evident in our studies with the murine equivalent. From these observations it is possible that differences in constitutive activity may explain the considerable differences in GABA potency reported between species observed by others and in this study. However, several other factors may also influence the observed potency shift, such as differences in relative subunit expression levels which may significantly impact on the final receptor stoichiometry. In particular, δ-containing receptors are prone to such variations in stoichiometry. This is evident from studies in which variations in the amount of RNA injected into *Xenopus* oocytes apparently yield different receptor populations [38]; likewise, concatemeric receptors also demonstrate that alternative assemblies of functional δ-containing receptors are possible [12,39]; and from reports that both high and low affinity conformations of the related α4β3δ receptor apparently exist [25,40].

In this study we report the first characterisation of specific GABA antagonists at α4β1δ receptors. The approximately 60-fold potency shift in the IC_50_ values for Zn^2+^ from low micromolar to mid nanomolar, when comparing α4β1δ and α4β1, is consistent with prior observations made for α4β3δ and α4β3 receptors [29]. Our determination of monophasic Zn^2+^ potency suggests it is highly unlikely that there is any contamination with binary α4β1 receptors in our α4β1δ-expressing oocytes. Also, the inability of Zn^2+^ to inhibit the response below the holding current baseline corroborates the absence of any constitutive activity caused by homomeric β1 receptors [41].

It has previously been suggested that the stoichiometry of binary GABA_A_ receptors containing only α and β subunits is dependent on the type of α and β subunit present, with a 3α:2β stoichiometry predominating for α1β2 receptors, but a 2α:3β stoichiometry preferred for α4β2 receptors [11]. The biphasic inhibitory behaviour of Zn^2+^ in our study would suggest the presence of a mixed GABA_A_ receptor population, perhaps with both 3α:2β and 2α:3β stoichiometries expressed in the oocyte. This conclusion is rationalized by the fact that an additional β subunit will contribute another H267 residue, known to be critical for Zn^2+^ inhibition [31], which may underpin the higher sensitivity component to Zn^2+^ in the biphasic inhibition curves.

This study also evaluated the pharmacology of additional compounds at α4β1δ known to interact with GABA_A_ receptors. We have established that the pharmacological profile of the β1-selective inhibitor SCS, at α4β1δ receptors, is in agreement with the values reported for α2β1γ2 receptors when expressed in *Xenopus* oocytes (IC_50_ = 4.36 nM and maximum inhibition of ~33% [34], suggesting that α4 and δ subunits do not strongly influence the molecular interaction with SCS.

Our data evaluating the inhibition of desensitized GABA responses by Cu^2+^ suggest that the maximal inhibition of Cu^2+^ is greater at α4β1δ (98% inhibition at 10 μM: expressed in oocytes) than at α6β3δ receptors (expressed in HEK293) where the maximal inhibition of the steady-state response was reported to be ~ 68% [33]. Although this difference may conceivably be due to the different expression systems, it is equally likely that it reflects some subunit-selectivity (α4 > α6) for Cu^2+^.

As hypothesized, THDOC concentrations at 30 nM and 100 nM were able to potentiate low (EC_7_) GABA concentrations, suggesting that α4β1δ receptors are likely to be modulated by physiologically relevant concentrations of neurosteroids, as akin to many other GABA_A_ receptor subtypes [35]. This is unsurprising given that the highly conserved neurosteroid binding site for potentiating GABA responses is contained within the receptor α subunit.

## Conclusions

In summary, we have extended the pharmacological profile of the α4β1δ receptor, a subtype that has been studied surprisingly little compared to its β2- and β3-containing counterparts, despite its presence in many significant brain areas. We report a high GABA potency (EC_50_ of 0.89 μM), a weaker sensitivity to Zn^2+^ compared to α4β1 receptors, and a high sensitivity to SCS and THDOC.

The observed discrepancy in potency compared to the human ortholog highlights the need for caution when making comparisons of pharmacological parameters across species for this particular GABA receptor subtype. For example, in addition to the different GABA potencies, there are notable differences in constitutive activity for this receptor isoform. Whether these differences translate into the native behaviour of this receptor remains to be determined. Finally, the different expression pattern of the β1 subunit compared to β2 and β3 subunits [24] suggests that β1-containing subtypes may offer novel therapeutic targets and opportunities, and emphasizes a greater need for subtype-selective ligands and/or genetically modified mice to better understand the native function and pharmacology of this GABA_A_ receptor isoform. Indeed recent findings suggest a clinically relevant role for β1-containing extrasynaptic GABA_A_ receptors in the nucleus accumbens mediating addictive behaviours related to excessive alcohol intake [23].

## Methods

Mouse α4 and β1 and rat δ cDNA were subcloned into a pRK5 expression vector using methods previously described [42]. γ-Aminobutyric acid (GABA), CuCl_2_ and tetrahydro-deoxycorticosterone (THDOC) were purchased from Sigma (Dorset, UK), ZnCl_2_ from BDP Biochemical (Poole, UK) and salicylidine salicylhydrazide (SCS) from Tocris (Bristol, UK).

### Expression of GABA subunits

Oocytes from *Xenopus laevis* toads were prepared as described [43]. Briefly, ovaries were removed using procedures approved by the UK Animals (Scientific Procedures) Act 1986 and local (UCL) ethics committee. To obtain free oocytes, ovarian lobes were cut into pieces and incubated in OR2 (85 mM NaCl, 5 mM HEPES, 1 mM MgCl_2_, pH adjusted to 7.6 with KOH) supplemented with 2-2.5 mg/mL collagenase type 1 (Lorne Laboratories, Danehill, UK) for 3-4 hrs. Oocytes were washed several times in OR2 and subsequently in Barth’s solution (88 mM NaCl, 1 mM KCl, 0.33 mM Ca(NO_3_)_2_, 0.41 mM CaCl_2_, 0.82 mM MgSO_4_, 2.4 mM NaHCO_3_, 10 mM HEPES, pH adjusted to 7.6 with NaOH) to isolate cells and remove follicular membranes. After preparation, the oocytes were injected with 27 or 55 nL of 30 ng/μL cDNA in a ratio of 1:1 (α4β1) or 1:1:5 (α4β1δ). Oocytes were subsequently maintained at 18°C in Barth’s solution supplemented with gentamycin (Invitrogen, Paisley, UK).

### Electrophysiological recordings

Recordings were performed at room temperature 2-4 days (α4β1) or 3-6 days (α4β1δ) post injection using two-electrode voltage-clamp (Axoclamp 2B amplifier, Digidata 1322A interface and pClamp 8 (Molecular Devices, Wokingham, UK)). Electrodes were filled with 3 M KCl and had resistances of 0.5 to 3 MΩ. The oocytes were clamped at a holding potential of -60 mV and were perfused with buffer (100 mM NaCl, 2 mM KCl, 2 mM CaCl_2_, 1 mM MgCl_2_, 5 mM HEPES, pH adjusted to 7.4 with NaOH) throughout the recordings. All currents were adjusted when necessary for voltage drop using equation 1:

1 $$ I={I}_{measured}\cdot \frac{\left({V}_h-{V}_{rev}\right)}{\left({V}_h-{V}_{rev}\right)+\varDelta V} $$

Where I_measured_ is the observed current, V_h_ is the holding potential (mV), V_rev_ is the Cl^−^ reversal potential for oocytes (usually ~ -30 mV), and *ΔV* is the change in the holding potential (mV) due to loss of clamp during at the peak of large GABA-evoked currents. Drugs were applied using a fast application system coupled to a small bath volume (~1 ml), enabling drug onset times of ~ 10 ms.

### Generation of concentration response curves

Agonist concentration response curves were generated by measuring peak responses normalized to a standard concentration of GABA applied between each or every second application (typically an EC_10-20_ concentration). To pool data from different oocytes, the data were fitted and normalized to the calculated peak of the curve using GraphPad Prism (version 6.0) according to equation 2;

2 $$ \frac{I}{I_{max}}=\frac{1}{1+{\left(\frac{E{C}_{50}}{\left[ GABA\right]}\right)}^n} $$

where I and I_max_ are the GABA activated current and maximal GABA current, respectively, and the EC_50_ is the GABA concentration inducing a half-maximal response and n is the Hill slope.

Zn^2+^ and SCS inhibition curves were generated by pre-application (for 60 s) of the inhibitor followed by co-applications of the inhibitor and GABA. For each co-application, the peak-response was measured. For Cu^2+^-inhibition curves, Cu^2+^ and GABA were co-applied and the steady-state response after 70 s was measured and normalized to a preceding GABA-application. All monophasic inhibition-curves were fitted to a modified version of equation 2. The biphasic Zn^2+^-inhibition curve was fitted to equation 3:

3 $$ {{\mathrm{I}}_{\mathrm{N}}}^{/}/\ {\mathrm{I}}_{\mathrm{N}} = \left[1\ \hbox{-} \mathrm{i}\ \right[{{\mathrm{B}}^{\mathrm{n}}}_{\mathrm{H}}/\ \left({{\mathrm{B}}^{\mathrm{n}}}_{\mathrm{H}} + \left({\mathrm{I}\mathrm{C}}_{50\mathrm{A}}{{}^{\mathrm{n}}}_{\mathrm{H}}\right)\right)\left]\hbox{-} \mathrm{j}\right[{{\mathrm{B}}^{\mathrm{n}}}_{\mathrm{H}}/\ \left({{\mathrm{B}}^{\mathrm{n}}}_{\mathrm{H}} + \left({\mathrm{I}\mathrm{C}}_{50\mathrm{B}}{{}^{\mathrm{n}}}_{\mathrm{H}}\right)\right)\left]\right] $$

where I_N_^/^ and I_N_ represent the normalised GABA-induced current in the presence and absence of Zn^2+^ at a concentration (B) respectively. (IC_50_)_A/B_ define the concentrations of Zn^2+^ producing 50% inhibition of the GABA-induced current and i and j represent weighting factors for the components A and B, where i + j = 1.

Potentiation by the neurosteroid THDOC was measured by co-application of GABA (EC_7_) and THDOC until a peak response was attained. Normalized responses were pooled and plotted as mean ± SEM from at least four different oocytes taken from at least two different *Xenopus* toads. For statistical evaluation, a repeated measures ANOVA with Dunnett’s post test was used. The significance level was set at *P < 0.05, **P < 0.01, ***P < 0.001. For inhibition curves, an F-test was performed to determine whether the data was either monophasic or biphasic.

## Abbreviations

GABA: γ-aminobutyric acid
HEK293: Human embryonic kidney 293
